# Refractory Renal Cell Cancer with Gastro-renal Fistula: A Rare Complication

**DOI:** 10.7759/cureus.6580

**Published:** 2020-01-06

**Authors:** Eric Tam, Elvira Neculiseanu, Gurinder Sidhu

**Affiliations:** 1 Medicine, State University of New York Downstate Medical Center, Brooklyn, USA

**Keywords:** kidney cancer, gastric fistula

## Abstract

Metastatic renal cell cancer is treated with systemic therapy, and cytoreductive nephrectomy can be offered in selected patients. The systemic therapy treatment options for kidney cancer have now expanded to include tyrosine kinase inhibitors, monoclonal antibodies, immunotherapy, and combinations thereof. Cytoreductive nephrectomy is considered a safe surgery in most patients. Patients with advanced kidney cancer are known to develop several paraneoplastic syndromes and malignant cachexia. We present the case of a patient with renal cell cancer who was treated with tyrosine kinase inhibitors and despite treatment her disease progressed with subsequent increase in the renal cancer, which led to the development of a fistula between the renal mass and the stomach.

## Introduction

Kidney cancers account for 3%-4% of all cancers in the United States. In the United States, there are approximately 65,000 new cases and 14,000 deaths from renal cell carcinoma (RCC) each year [1]. While kidney cancer is typically a disease of the elderly, it is increasingly being diagnosed in younger patients. Most patients with RCC present with an incidental radiographically detected mass. The symptoms of flank mass, weight loss, and hematuria are typically associated with advanced disease. Radiological assessment of a renal mass involves pre- and post-contrast computed tomography or magnetic resonance imaging. Most cases of RCC are sporadic, but approximately 5% can be associated with hereditary kidney cancer syndromes [2]. Clear cell carcinoma is the most common pathological subtype [3]. 

Nearly half of the patients with RCC present with a small renal mass and are surgically treated, with a partial nephrectomy. If partial nephrectomy is not possible, then radical nephrectomy is the treatment of choice. Cytoreductive nephrectomy (CN) followed by systemic therapy is the standard treatment of advanced RCC in patients with oligometastatic disease, good performance status, and good prognostic features [4]. This approach has been accepted by the National Comprehensive Cancer Network in its guidelines on the management of metastatic RCC [5]. This multimodality treatment approach has improved progression-free survival and overall survival. Advanced RCC is known to be associated with several paraneoplastic syndromes such as anemia, fever, thrombocytosis, hypercalcemia, cachexia, and hepatic dysfunction.

## Case presentation

**Figure 1:**
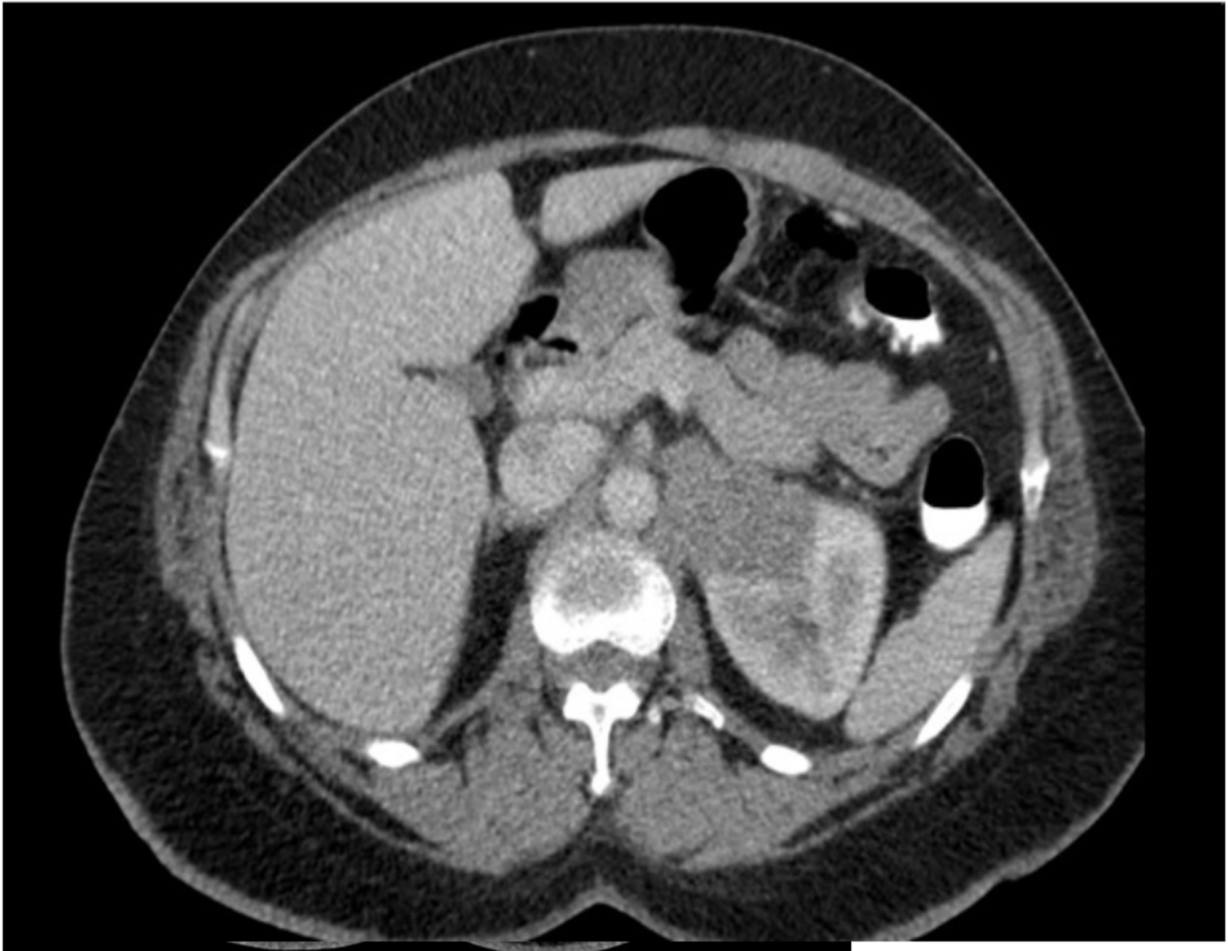
Contrast-enhanced CT scan of the abdomen showing left kidney mass at initial presentation.

A 56-year-old woman with well-controlled hypertension presented with 20 lbs weight loss over the preceding three months, and on workup was found to have a large left renal mass in the renal hilum with multiple regional lymph node enlargement and bilateral pulmonary nodules (Figure 1). 

**Figure 2:**
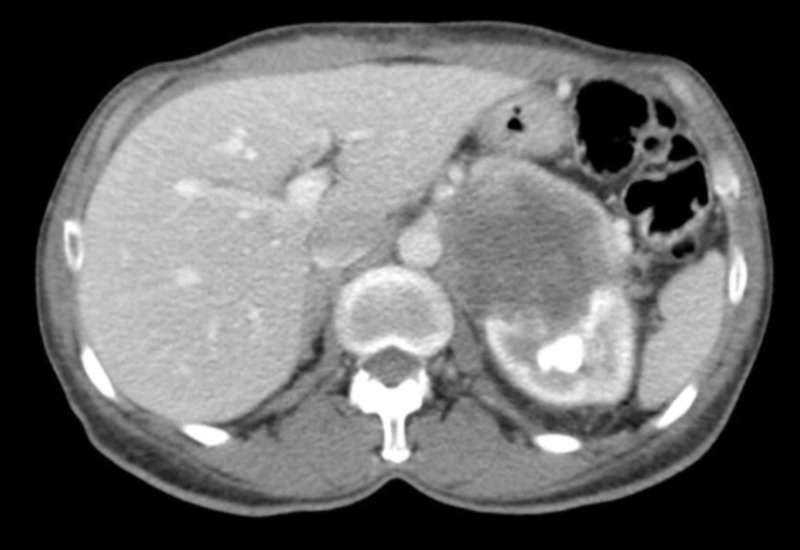
Contrast-enhanced CT scan of the abdomen showing progression of the left kidney cancer.

Her lab testing was significant for anemia with a hemoglobin of 9.1 g/dL. Her Karnofsky Performance Status score was 50. Renal and liver functions were normal, and calcium level was within the normal limits. Percutaneous biopsy of the renal mass was consistent with RCC with clear cell histology and no sarcomatoid variant. Using the Memorial Sloan Kettering Cancer Center (MKSCC) prognostic model for kidney cancer, she was found to be in the poor risk group. She was not offered CN and was started on systemic therapy with sunitinib. She tolerated sunitinib well and was compliant, but despite four months of systemic treatment, she continued to lose weight and on repeat imaging, the renal mass was shown to be enlarging, suggesting refractory disease (Figure 2). 

**Figure 3:**
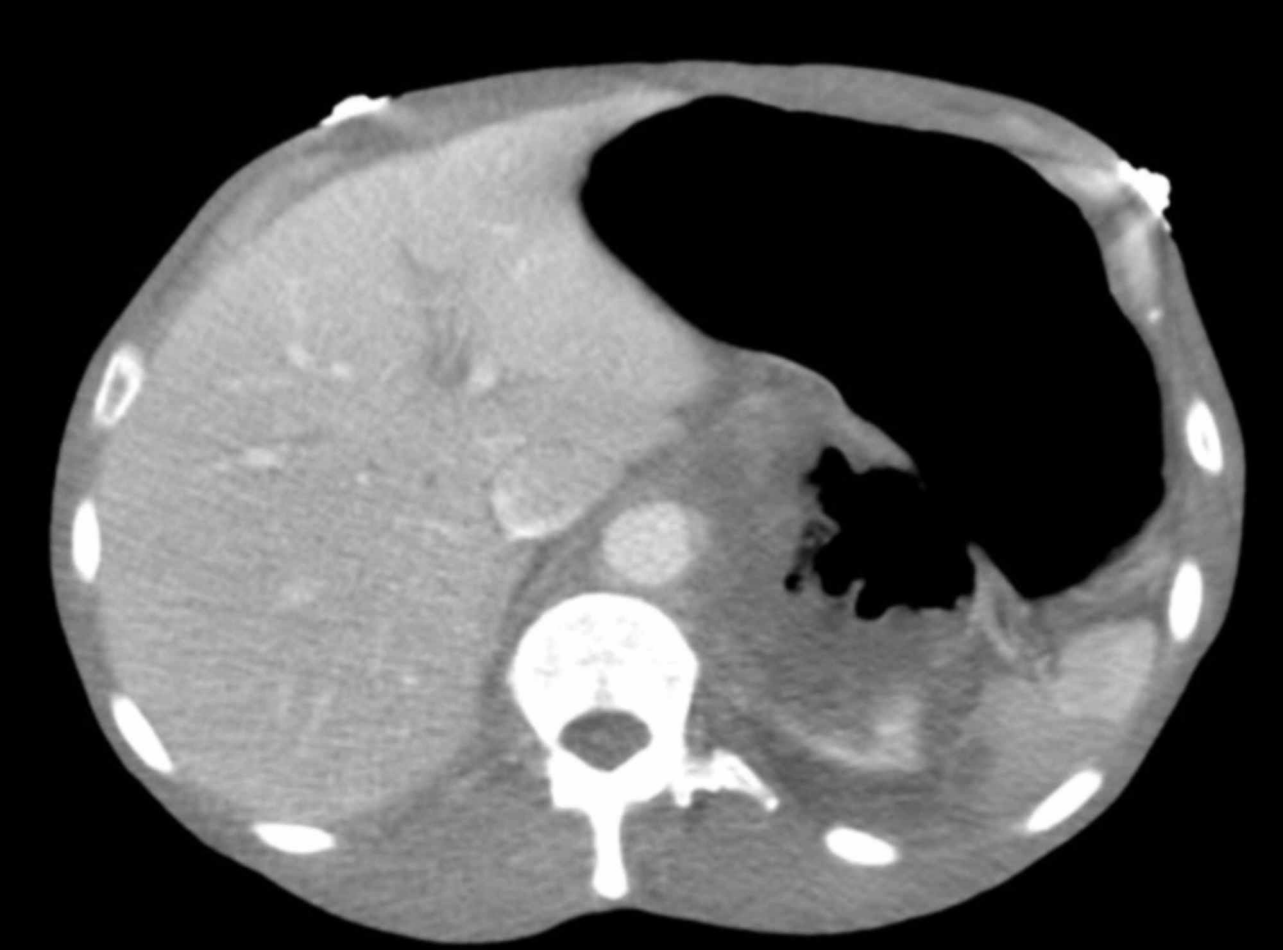
Pre-contrast CT scan of the abdomen with stomach dilation, increase in renal mass, and presence of fistula between stomach and kidney mass.

Her treatment was switched to temsirolimus and after two months of treatment, she presented to the emergency department with complaints of abdominal pain and distention, nausea, and vomiting of three days of duration. Repeat imaging indicated massive gastric distention and an increased size of the previously seen renal mass with a substantial central necrotic component (Figure 3).

**Figure 4:**
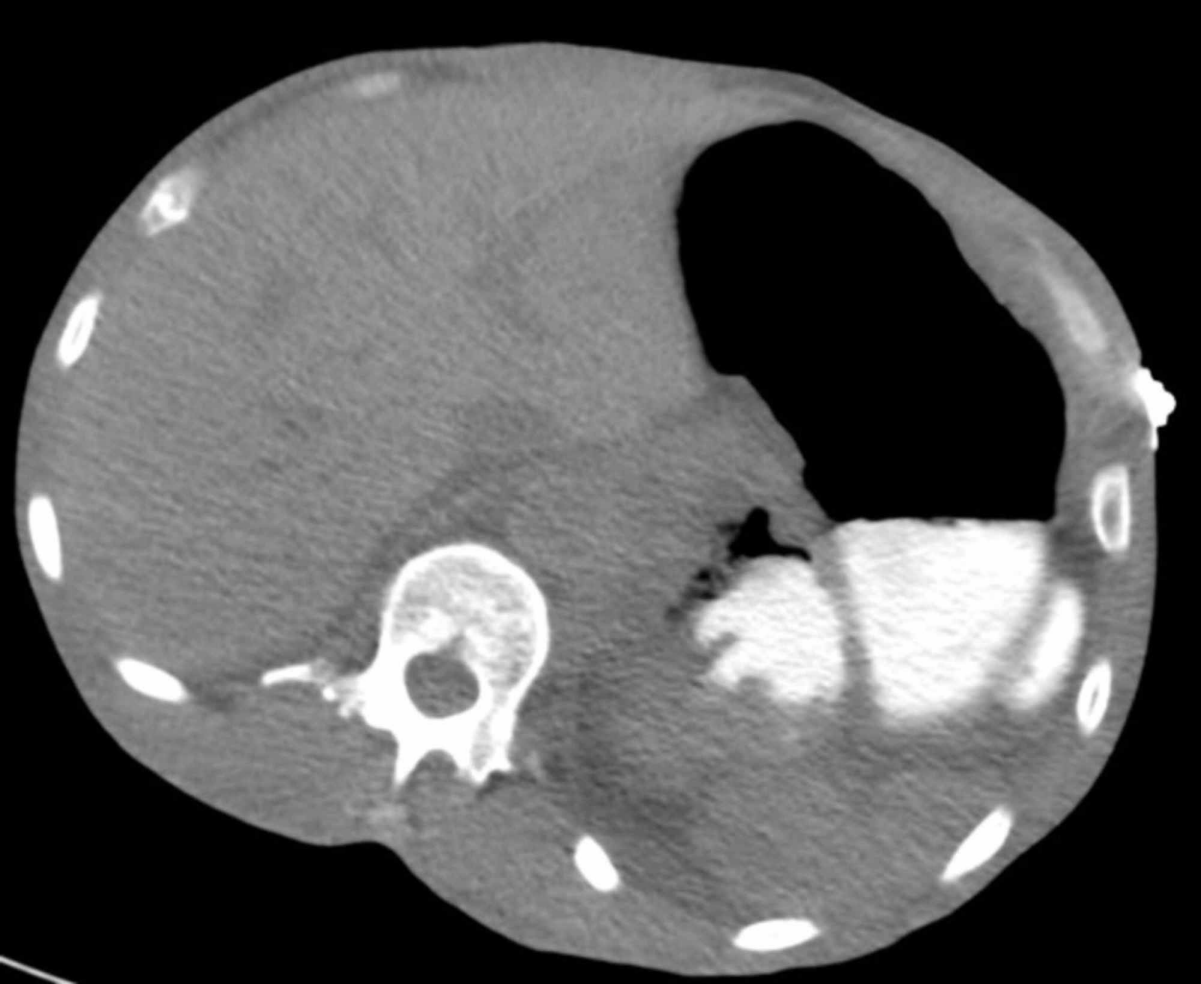
Contrast CT of the abdomen with large necrotic renal mass and presence of oral contrast in the necrotic renal mass.

There was new direct infiltration of the renal mass into the stomach. Oral contrast was noted to extravasate from the stomach to the necrotic renal mass (Figure 4). 

The patient was unable to tolerate oral diet and opted for comfort care. She died in hospice care one week later.

## Discussion

Fistula formation between the kidney and gastrointestinal tract is uncommon, with most cases reported as renocolic fistulas [6]. Renoalimentary fistulas are often associated with infection, ischemia, or necrosis precipitated by an underlying condition such as nephrolithiasis, trauma, or iatrogenic interventions such as radiofrequency ablation and cryoablation [7]. Diagnosis is usually by barium enema or computed tomography with contrast. Intravenous pyelography may provide limited diagnostic benefit as the affected kidney may not have adequate function.

Systemic therapy for metastatic RCC includes tyrosine kinase inhibitors (TKI), immunotherapy, or a combination of both [8]. Each treatment is adjusted to patient individually, using MSKCC or International Metastatic Renal Cancer Database Consortium risk group stratification. The new therapies significantly increase disease-free survival and improve patient quality of life. Sunitinib is the preferred option for first-line treatment of patients with medically unresectable clear cell metastatic RCC with poor/intermediate risk features. The fast evolving field in treatment of malignancies is immunotherapy. Recently, FDA approved the combination of nivolumab and ipilumamab as a first-line therapy for patients with intermediate/poor risk [9]. Temsorolimus is also an option for poor risk patient [10]. 

CN has been the standard of care for metastatic RCC prior to systemic therapy and still recommended in patient with potentially surgically resectable primary tumor or oligometastatic disease. Retrospective studies conducted in TKI era still suggested that CN could be beneficial and patients with good PS, good risk group, and oligometastatic disease. Long-term relapse free survival was reported in these patients [11-13]. 

In the setting of progressive local renal tumor, the role of palliative nephrectomy needs to be assessed carefully in each patient. 

In our patient, her gastro-renal fistula was likely a result of necrosis caused by progressive tumor growth. This case is the first reported local complication of RCC leading to the formation of a gastro-renal fistula with a fatal outcome. CN is known to extend overall survival in the pre- and post-TKI era [11-13]. In this case, had the patient undergone CN, it might have also reduced the likelihood of morbidity from a progressively enlarging RCC.

## Conclusions

CN is a standard treatment of metastatic RCC in selected patients. It is a safe and effective procedure for most patients. Progressive metastatic RCC is associated with several systemic syndromes. Systemic treatment options for RCC have expanded in the last several years. The case presented illustrates the potential benefits of CN in reducing local complications of RCC. The morbidity and mortality of progressive RCC can potentially be avoided by having a patient undergo CN at initial diagnosis of metastatic RCC. Surgery to treat local complications of RCC later in the course of the disease can have significantly more morbidity and given the patients are usually in a worse overall condition, it is frequently not possible. 

## References

[1] Siegel R, Naishadham D, Jemal A (2012). Cancer statistics. CA Cancer J Clin.

[2] Popova T, Hebert L, Jacquemin V (2013). Germline BAP1 mutations predispose to renal cell carcinomas. Am J Hum Genet.

[3] Cohen AJ, Li FP, Berg S (1979). Hereditary renal-cell carcinoma a ssociated with a chromosomal translocation. N Engl J Med.

[4] Culp SH, Tannir NM, Abel EJ (2010). Can we better select patients with metastatic renal cell carcinoma for cytoreductive nephrectomy?. Cancer.

[5] Motzer RJ, Agarwal N, Beard C (2011). Kidney cancer. J Natl Compr Canc Netw.

[6] Blatstein LM, Ginsberg PC (1996). Spontaneous renocolic fistula: a rare occurrence associated with renal cell carcinoma. J Am Osteopath Assoc.

[7] de Arruda HO, Goldman S, Andreoni C, Maia RS, Szejnfeld J, Ortiz V (2006). Renoduodenal fistula after renal tumor ablation with radiofrequency. Surg Laparosc Endosc Percutan Tech.

[8] Motzer RJ, Michaelson MD, Redman BG (2006). Activity of SU11248, a multitargeted inhibitor of vascular endothelial growth factor receptor and platelet-derived growth factor receptor, in patients with metastatic renal cell carcinoma. J Clin Oncol.

[9] Motzer RJ, Tannir NM, McDermott DF (2018). Nivolumab plus ipilimumab versus sunitinib in advanced renal-cell carcinoma. N Engl J Med.

[10] Hudes G, Carducci M, Tomczak P (2007). Temsirolimus, interferon alfa, or both for advanced renal-cell carcinoma. N Engl J Med.

[11] Choueiri TK, Xie W, Kollmannsberger C (2011). The impact of cytoreductive nephrectomy on survival of patients with metastatic renal cell carcinoma receiving vascular endothelial growth factor targeted therapy. J Urol.

[12] Méjean A, Ravaud A, Thezenas S (2018). Sunitinib alone or after nephrectomy in metastatic renal-cell carcinoma. N Engl J Med.

[13] Motzer RJ, Russo P (2018). Cytoreductive nephrectomy: patient selection Is key. N Engl J Med.

